# Cutaneous Pseudoepitheliomatous Hyperplasia from a Displaced Metallic Orthopedic Implant

**DOI:** 10.1155/2022/9139213

**Published:** 2022-04-07

**Authors:** Franklin R. Blum, Logan S. D'Souza

**Affiliations:** ^1^UNC Chapel Hill School of Medicine, Chapel Hill, NC, USA; ^2^UNC School of Medicine Asheville, Asheville, NC, USA

## Abstract

While rare, cutaneous SCC in patients with darker Fitzpatrick skin types is essential to identify and investigate early and can have a myriad of clinical presentations. While clinical history-taking of suspicious skin lesions is often symptom-driven, other key patient history components, such as surgical history, are often overlooked. Differentiating, prioritizing, and risk-stratifying hyperkeratotic, verrucous papules in patients with darker Fitzpatrick skin types is an essential clinical skill for clinicians to develop to serve an increasingly diverse patient population. This original report presents the case of a displaced orthopedic screw causing pseudoepitheliomatous hyperplasia that was initially misdiagnosed as squamous cell carcinoma. This case highlights the importance of careful consideration of surgical history, choice of biopsy method, and skin type when examining lesions concerning for squamous cell carcinoma.

## 1. Introduction

The differential of a cutaneous verrucous lesion is broad, but includes verruca vulgaris, verrucous carcinoma, pseudoepitheliomatous hyperplasia (PEH), and squamous cell carcinoma (SCC). On physical exam, such lesions can have significant morphological overlap, requiring histologic differentiation for definitive diagnosis. This report presents the case of a metallic foreign body causing PEH that was initially misdiagnosed as squamous cell carcinoma.

Pseudoepitheliomatous hyperplasia is a benign proliferation of squamous epithelium that can extend into the dermis [1]; it is closely related to chronic inflammation of the underlying dermis. Identified causes of PEH include malignancy, wounds, retained foreign material, and even tattoo pigments [1, 2]. Considered benign, PEH can be difficult to distinguish from other hyperproliferative skin disorders on exam, especially SCC. The term pseudocarcinomatous hyperplasia is used by some for PEH for this malignant association [3]. Histologically, PEH and SCC can be difficult to differentiate, but factors that favor SCC include abundant mitoses, nuclear atypia, necrotic keratoses, and deep connective tissue invasion by epithelium [4]. Currently, there are no reports of cutaneous PEH being caused from a displaced metal orthopedic implant, the topic of this account, making this likely the first report.

Squamous cell carcinoma and PEH can be challenging to differentiate clinically in darker Fitzpatrick skin types. Nonmelanoma skin cancer is exceedingly rare in the Black population, with an estimated incidence of approximately 3 per 100,000 population [5], as compared to over 230 per 100,000 population in Whites [6]. Coupled with this low incidence, skin cancer appearance can also vary. In this case, the patient's young age, darker Fitzpatrick skin type, and the lesion's morphology and location of low sun exposure placed him at high concern for skin cancer, necessitating urgent dermatologic exploration.

## 2. Case Synopsis

**Figure 1:**
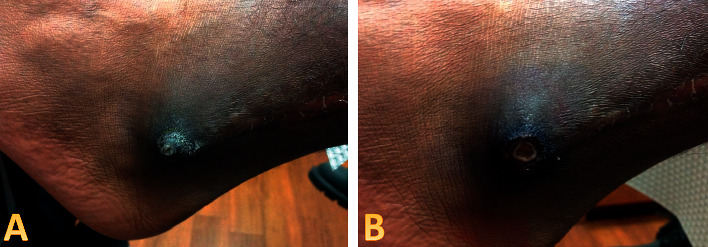
Initial clinic evaluation. 1.6 × 1.6 cm irregular, hyperkeratotic, verrucous-appearing papule distributed on the left lateral malleolus. A surgical scar is visualized, running vertically on the lateral ankle superior to the lesion (a). A biopsy by shave was performed (b).

**Figure 2:**
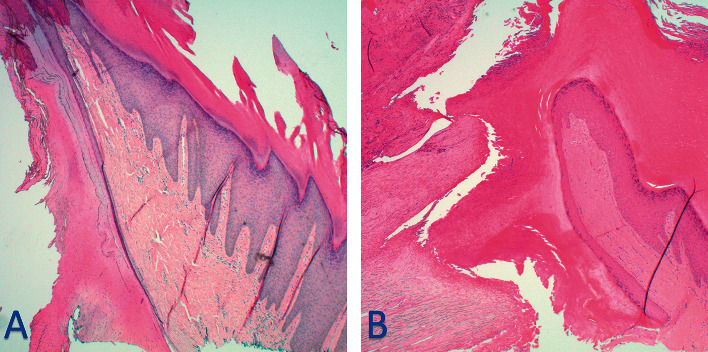
Histopathological imaging from initial biopsy (a) and 6-month recurrence (b). Papillomatous and endophytic squamous proliferation is present with keratin plug, parakeratin, and mild pleomorphism of keratinocytes extending to the base of the biopsy (a). 6 months later, the biopsy showed overlying thickened parakeratin alternating with parakeratin with serum accumulation (b). Collections of neutrophils were also observed in the stratum corneum (b), so a PAS stain was performed to evaluate for the presence of fungal organisms. No fungal organisms were noted. The adjacent *epidermis* displayed acanthosis and papillomatosis composed of suprabasilar keratinocytes without significant atypia (b).

A 22-year-old Fitzpatrick type 5 male presented to dermatology with a chief complaint of a growing and painful skin lesion on the left ankle for several months at the base of a fibular fracture scar from four years prior. As he was a college football player, the lesion was especially painful when moving laterally. Upon initial inspection by a physician assistant, a 1.6 cm × 1.6 cm hyperkeratotic, verrucous-appearing plaque was identified on the left lateral malleolus at the inferior end of a vertical linear scar (Figure 1(a)). A biopsy by shave method was performed (Figure 1(b)), which showed atypical squamous proliferation worrisome for invasive, well-differentiated squamous cell carcinoma (Figure 2(a)). He returned to clinic one week later, at which time electro-desiccation and curettage were performed. A timeline of events for his clinical care is listed in Table 1.

**Figure 3:**
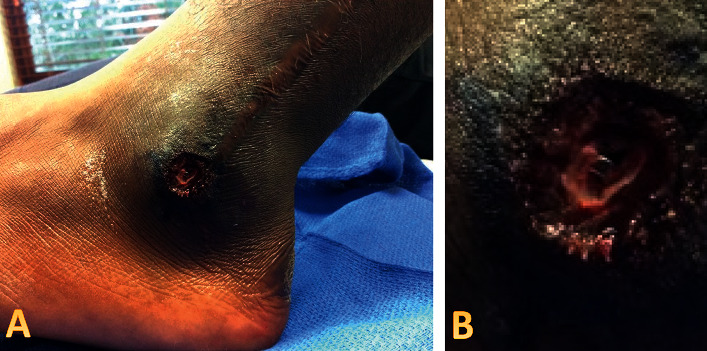
Mohs intraoperative imaging. After debulking, a metallic foreign body is present centrally (a), most consistent with a displaced orthopedic surgical screw. A closer view is appreciated (b).

However, treatment was unsuccessful, as the patient returned to the clinic six months later reporting a one-month history of a similar lesion worrisome for recurrence. He was seen by a different provider from his initial evaluation. As the patient had professional football aspirations, the lesion was debulked and biopsied that same day. Biopsy could not exclude an atypical process (Figure 2(b)). A periodic acid–Schiff (PAS) stain was performed to exclude a fungal process and returned negative. Definitive treatment was set for the conclusion of the collegiate season a few weeks later. Further complicating his timeline was the upcoming National Football League (NFL) draft, an annual event during which professional football teams select new players for their rosters. He opted for Mohs surgery for a higher cure rate to minimize normal tissue removal and to prioritize his need to heal quickly for training. During evaluation on the day of his Mohs surgery, the recurrent lesion was visualized (Figure 3), and an operative plan was made. Upon shave debulking before the first stage of tumor extirpation, a shiny, somewhat mobile metal screw was visualized centrally (Figure 3).

The metallic object identified was most consistent with a displaced orthopedic surgical screw, concerning for pending implant failure. Mohs surgery was aborted given this new correlative clinical information. The patient verified that the linear scar on his lateral ankle was from a prior orthopedic surgical repair of a fibular fracture that was repaired with metal screws and plates approximately four years prior. It was posited that repetitive, forceful, high-intensity football training likely stressed the orthopedic fracture repair and contributed to failure, explaining the delayed lesion onset. The debulk was sent to dermatopathology for consultation and comparison with past biopsies. With additional tissue and clinical information, dermatopathology's findings were most consistent with cutaneous pseudoepitheliomatous hyperplasia. The patient was referred out of state to the original orthopedic surgeon who performed the corrective surgery four years prior before addressing the displaced metal implant, the underlying cause of his cutaneous proliferation. No adverse events were reported in the years following. The patient was subsequently lost to follow-up.

## 3. Discussion

While the underlying metallic foreign body and unique pathological features gave the definitive diagnosis, differentiating between benign and malignant causes of a verrucous papule requires a breadth of consideration. Lesion location, duration, and appearance as well as patient age, sex, race, and other demographic information influence a physician's clinical lens for diagnosis and management. However, one factor that infrequently plays a diagnostic role in dermatology is past surgical history. Reviewing a patient's prior surgical history, not just limited to dermatologic surgeries, should be performed preoperatively [7]. In this case, prior surgical history was vital to final diagnosis, and overlooking this fact was a fault in care that may have resulted in undue burden for the patient and medical system.

A limitation to our clinical approach that possibly resulted in unnecessary interventions and delayed diagnosis was inadequate lesion biopsy. Importantly, as described by Zayour et al., PEH may be misinterpreted as SCC in cases where the primary process localized in the dermis is not readily apparent or the biopsy is superficial and lacks a sufficient portion of the dermis [1]. A biopsy by shave method was performed at the initial evaluation, which may not have been sufficiently deep. Although guidelines of care published in the Journal of the American Academy of Dermatology (JAAD) do not indicate an optimal biopsy method for lesions concerning for SCC [8], National Comprehensive Cancer Network (NCCN) guidelines advise, when taking a biopsy, “if more than superficial lesion, inclusion of deep reticular dermis preferred” [9]. This case report challenges the JAAD guidelines and supports the more detailed NCCN guidelines. A biopsy by punch or surgical biopsy would have included the deeper reticular dermis.

Another important factor for consideration in this case was the patient's skin type. While rare, cutaneous SCC in patients with darker Fitzpatrick skin types is essential to identify and investigate early. The most important risk factor for SCC in this population is chronic scarring processes and areas of chronic inflammation [5], with common causes being leg ulcers, burn scars, radiation dermatitis, and discoid lupus [5, 10]. Of notable concern, cutaneous SCC in Black patients has been associated with a radically high mortality rate of around 18.4% [11]. Frequently, these tumors present at a more advanced stage and thus worse prognosis compared to patients with lighter skin types [12]. Additionally, cutaneous SCC arising from chronic scarring or inflammation in Black patients carries a metastatic risk of 20–40% compared to 1–4% metastatic risk of sun-induced SCC in Whites [5]. In general, nonhealing nodules adjacent to scarring necessitate biopsy to exclude malignancy [5]. As the lesion in this case was found within a previous surgical scar, urgent evaluation was merited. Although a benign case of PEH was identified, comparable lesion presentations should be treated with the same level of urgency in this patient population.

There is a paucity of literature describing cases of PEH in patients of darker skin types. Specifically, using combinations of the search terms “pseudo-epitheliomatous hyperplasia,” “black,” “skin,” “African American,” “Fitzpatrick 4,” and “Fitzpatrick 5” on PubMed, only one pathology review had case descriptions of PEH in patients with darker skin types [13] and one discussed components of PEH in conjunction with verrucous sarcoidosis [14], but not from a metallic foreign body. Furthermore, using the search terms “metal,” “screw,” “foreign body,” “pseudo-epitheliomatous hyperplasia,” “orthopedic,” “surgery,” and “implant” on PubMed yielded reports of PEH from intentional mercery injection [15, 16] and unidentified mercury contact [17]. However, no reports were found of cutaneous PEH caused from a displaced metal orthopedic implant, making this likely the first report.

Lastly, in patients with a verrucous papule presenting in close proximity to or within a known prior orthopedic surgical scar, considering preoperative imaging may reduce unnecessary patient and health system burden. This patient had a concerning lesion overlying previously placed metallic orthopedic implants. X-ray investigation may have revealed the underlying etiology of the lesion earlier than with dermatologic investigation alone.

## 4. Conclusion

This is likely the first report of PED caused from a displaced metal orthopedic implant, which occurred in a patient with Fitzpatrick type 5 skin. Differentiating between PEH and SCC in patients with darker Fitzpatrick skin types presents a clinical and histopathological challenge that carries significant risk if an incorrect diagnosis is made. This case highlights the importance of a careful consideration of a patient's surgical history, skin type, and choice of biopsy method when investigating lesions concerning for squamous cell carcinoma. Arriving at the correct diagnosis early in a lesion's treatment course can prevent undue burden on patients and the greater health system.

## Figures and Tables

**Table 1:** Timeline of events.

Event	Finding(s)	Intervention(s)
Initial evaluation by PA	Lesion identified on the left lateral malleolus	Shave biopsy performed
*1 week later*		
Treatment with 1^st^ MD	Biopsied lesion	ED&C^*∗*^ with pathology

*6 months later*		
Reevaluation with 1^st^ MD	Concern for recurrent lesion	Debulk with pathology performed definitive treatment scheduled

*3 weeks later*		
Definitive treatment with 2^nd^ MD	Recurrent lesion	Mohs surgery

*Intraoperation*		
Surgery cessation	Discovery of displaced metallic orthopedic implant	Referral to orthopedics

^*∗*^Electrodessication and curettage; PA = physician associate; MD = medical doctor.

## Data Availability

There is no data involved in this report.

## Abbreviations

PEH: Pseudoepitheliomatous hyperplasia
SCC: Squamous cell carcinoma.

## References

[1] Zayour M., Lazova R. (2011). Pseudoepitheliomatous hyperplasia: a review. *The American Journal of Dermatopathology*.

[2] Balfour E., Olhoffer I., Leffell D., Handerson T. (2003). Massive pseudo-epitheliomatous hyperplasia: an unusual reaction to a tattoo. *The American Journal of Dermatopathology*.

[3] Valdebran M., Neuhaus I., North J. (2017). Pseudocarcinomatous epithelial hyperplasia induced by imiquimod: a mimic of cutaneous squamous cell carcinoma. *Dermatologic Surgery*.

[4] Nayak V. N., Uma K., Girish H. C., Murgod S., Shyamala K., Naik R. B. (2015). Pseudoepitheliomatous hyperplasia in oral lesions: a review. *Journal of International Oral Health*.

[5] Gloster H. M., Neal K. (2006). Skin cancer in skin of color. *Journal of the American Academy of Dermatology*.

[6] Scotto J., Fears T. R., Fraumeni J. F. (1983). *Incidence of Nonmelanoma Skin Cancer in the United States*.

[7] Otley C. C. (2006). Perioperative evaluation and management in dermatologic surgery. *Journal of the American Academy of Dermatology*.

[8] Kim J. Y. S., Kozlow J. H., Mittal B., Moyer J., Thomas O., Phillip R. (2018). Guidelines of care for the management of cutaneous squamous cell carcinoma. *Journal of the American Academy of Dermatology*.

[9] National Comprehensive Cancer Network (2022). Squamous cell skin cancer. https://www.nccn.org/professionals/physician_gls/pdf/squamous.pdf.

[10] Que S. K. T., Zwald F. O., Schmults C. D. (2018). Cutaneous squamous cell carcinoma. *Journal of the American Academy of Dermatology*.

[11] Mora R. G., Perniciaro C. (1981). Cancer of the skin in blacks. I. A review of 163 black patients with cutaneous squamous cell carcinoma. *Journal of the American Academy of Dermatology*.

[12] Hu S., Soza-Vento R. M., Parker D. F., Kirsner R. S. (2006). Comparison of stage at diagnosis of melanoma among Hispanic, black, and white patients in Miami-Dade County, Florida. *Archives of Dermatology*.

[13] Lynch J. M. (2004). Understanding pseudoepitheliomatous hyperplasia. *Pathology Case Reviews*.

[14] Sussman M. E., Pousti B. T., Grossman S. K., Lee J. B., Hsu S. (2021). Verrucous sarcoidosis: a rare clinical presentation of sarcoidosis. *Cureus*.

[15] Lupton G. P., Kao G. F., Johnson F. B., Graham J. H., Helwig E. B. (1985). Cutaneous mercury granuloma. A clinicopathologic study and review of the literature. *Journal of the American Academy of Dermatology*.

[16] Vernon S. E. (2005). Case report: subcutaneous elemental mercury injection—clinical observations and implications for tissue disposal from the histopathology laboratory. *Annals of Clinical and Laboratory Science*.

[17] Bothale K., Dongre T., Mahore S., Pande S. (2013). Cutaneous mercury granuloma. *Indian Journal of Occupational and Environmental Medicine*.

